# RETRACTION: ING5‐Mediated Antineuroblastoma Effects of Suberoylanilide Hydroxamic Acid

**DOI:** 10.1002/cam4.70849

**Published:** 2025-04-04

## Abstract

**RETRACTION**: J.‐C. Wu, H.‐M. Jiang, X.‐H. Yang and H.‐C. Zheng, “ING5‐Mediated Antineuroblastoma Effects of Suberoylanilide Hydroxamic Acid,” *Cancer Medicine* 7, no. 9 (2018): 4554–4569, https://doi.org/10.1002/cam4.1634.

The above article, published online on 09 August 2018 in Wiley Online Library (wileyonlinelibrary.com), has been retracted by agreement between the journal Editor‐in‐Chief, Stephen Tait; and John Wiley & Sons Ltd. The retraction has been agreed upon due to the duplication of several protein bands in Figures 1H, 2H, and 5C, which were found in previously published articles by some of the same authors. The authors contacted the journal to explain that the errors were inadvertent and occurred because the studies were conducted simultaneously in the same laboratory; they also provided some data for evaluation. The authors state that the main conclusions of this manuscript are unaffected, but the extent and nature of the duplications have significantly undermined the editor's confidence in the validity of the results presented. The authors disagree with the retraction.

**RETRACTION**: J.‐C. Wu, H.‐M. Jiang, X.‐H. Yang and H.‐C. Zheng, “ING5‐Mediated Antineuroblastoma Effects of Suberoylanilide Hydroxamic Acid,” *Cancer Medicine* 7, no. 9 (2018): 4554–4569, https://doi.org/10.1002/cam4.1634.

The above article, published online on 09 August 2018 in Wiley Online Library (wileyonlinelibrary.com), has been retracted by agreement between the journal Editor‐in‐Chief, Stephen Tait; and John Wiley & Sons Ltd. The retraction has been agreed upon due to the duplication of several protein bands in Figures 1H, 2H, and 5C, which were found in previously published articles by some of the same authors. The authors contacted the journal to explain that the errors were inadvertent and occurred because the studies were conducted simultaneously in the same laboratory; they also provided some data for evaluation. The authors state that the main conclusions of this manuscript are unaffected, but the extent and nature of the duplications have significantly undermined the editor's confidence in the validity of the results presented. The authors disagree with the retraction.

